# The Genetic Diversity of *Bletilla* spp. Based on SLAF-seq and Oligo-FISH

**DOI:** 10.3390/genes13071118

**Published:** 2022-06-22

**Authors:** Jie Huan, Zhoujian He, Yuting Lei, Wenjun Li, Liqiong Jiang, Xiaomei Luo

**Affiliations:** 1 College of Forestry, Sichuan Agricultural University, Huimin Road 211, Wenjiang District, Chengdu 611130, China; 15388135911@163.com (J.H.); hezhouj@163.com (Z.H.); mihualyt@126.com (Y.L.); 2 Institute of Forestry, Chengdu Academy of Agriculture and Forestry Sciences, Nongke Road 200, Wenjiang District, Chengdu 611130, China; lwj13980429970@163.com (W.L.); liqiong_jiang@163.com (L.J.)

**Keywords:** *Bletilla*, oligo-FISH, SLAF, molecular cytogenetics

## Abstract

*Bletilla* spp. Rchb. F. is a traditional Chinese medicinal material. In this study, *Bletilla striata* (Thunb. ex A. Murray) Rchb F, *Bletilla formosana* (Hayata) Schltr, and *Bletilla ochracea* Schltr were collected to analyze the genetic diversity of 16 materials using specific site-amplified fragment sequencing (SLAF-seq) and fluorescence in situ hybridization (FISH). The results showed that the phylogenetic tree of the single-nucleotide polymorphism (SNP) data rendering system was correlated with the shape and geographical distribution of the material. The results of the population structural analysis showed that all the materials containing yellow labellum came from the same ancestor. The results of the principal component analysis were able to preliminarily judge the genetic distance and provided a reference for the selection of hybrid parents. The FISH analysis showed that the chromosomes of *B. striata* were 2n = 32 and the chromosomes of the *B. striata* (safflower) mutant were 2n = 34 and the chromosomes of *B. ochracea* and *B. formosana* were 2n = 34–36. The (AG_3_T_3_)_3_ non-terminal signal was different from the 5S rDNA signal. These results revealed that the 16 materials had rich genetic diversity, which can provide molecular and cytogenetic data for the study of the genus and its relatives and serve as a reference for the breeding of new genus varieties and improve breeding efficiency and cost.

## 1. Introduction

*Bletilla* spp. Rchb. F. is a perennial orchid herb that is usually purple or yellow [1]. There are six species of *Bletilla* in the world, and they are widely distributed in northern Myanmar, Japan, and China. There are four species in China [2]: *B. striata* (Thunb. ex A. Murray) Rchb F. (hyacinth orchid), *B. formosana* (Hayata) Schltr. (Taiwan ground orchid)*, B. ochracea* Schltr. (Chinese butterfly orchid), and *Bletilla sinensis* (Rolf) Schltr. *Bletilla* has medicinal [3] and ornamental value [4,5], and *Bletilla* polysaccharides are used in medical materials [6,7,8,9,10], health foods [11], and whitening and skin care products [12,13]. *Bletilla* resources have been over-collected and are on the verge of extinction, and its medicinal sources are becoming exhausted [14]. Therefore, it is urgent to protect and define the fine germplasm resources of *Bletilla* [15].

Molecular markers based on genetic material are a direct reflection of DNA information and are not affected by the developmental stage and state of plants, growing environment, organ sampling location, etc. [16]. They have high stability and reliability, and sampling does not affect plant growth [17]. Single-nucleotide polymorphism (SNP) manifests abundant polymorphism based on the minimal structural single-base differences in genetic variations in genomic DNA. For example, the transition or transversion of functional gene combination of a single base, the insertion of functional gene combination of a base, and the deletion of base gene mutation [18] directly reflect the new genetic marking methods of the differences in the DNA level between individuals of different organisms. SNP directly reflects the new genetic markers of the differences in DNA level between individuals of different organisms to distinguish different varieties and study the genetic relationships between varieties [19]. Specific locus-amplified fragment sequencing (SLAF-seq) [20,21] using the restriction endonuclease (RE) is used to interrupt the genome to reduce its complexity, and then high-throughput sequencing is performed on specific segments of the genome to obtain information sequences that can replace the whole genome of the target species. It has the advantages of the simple and rapid acquisition of the SNP markers and genotypes covering the whole genome [22,23]. Compared with traditional methods, it has the characteristics of a flexible scheme, the ability to avoid repeated sequences, and the ability to realize the development of SNP-specific molecular markers in nonparametric genome species [24]. At present, it has been applied in many plants, such as *Melocalamus arrectus* Yi. [25], *Humulus lupulus* Linn. [26], *Allium cepa* L. [27], and *Glycine max* (L.) Merr. [28].

Considering the number of chromosomes is a useful way to distinguish between species. FISH has widely been used to measure the genetic relationships among plant species [29,30], phylogenetic reconstruction [31], and homologous chromosome identification [32,33], and the results are reliable and fast [34]. Chromosome data are useful for species classification and the basis of cytogenetics. Through karyotyping between *Bletilla*, information about the origin, phylogeny, genetic breeding, and variety improvements in different species can be learned [35,36]. The use of oligonucleotide systems enabled the first comprehensive cytogenetic analysis of *Bletilla* in the genomics era. A combination of 5S rDNA, and (AG_3_T_3_)_3_ probes has been used in *Hibiscus mutabilis* L. [37], *Hippophae rhamnoides* L. [38], *Chimonanthus campanulatus* R. H. Chang & C. S. Ding [39], *Juglans regia* L. and *J. sigillata* Dode. [40]. 

In the present study, FISH was used for the karyotype analysis of *Bletilla* using 5S rDNA and (AG_3_T_3_)_3_ probes, which facilitated the counting of chromosomes of *Bletilla* and the comparison of genetic relationships among genera. Specific fragments of the whole genome of *Bletilla* were obtained via SLAF, and SNP markers were developed. Through the asymmetry and ploidy of the karyotype and signal, we can judge the evolutionary degree and genetic relationship of plant materials, as well as the genetic distance [41,42]. Our study provides molecular cytogenetic data for the study of genetic diversity and benefits for further breeding studies on *Bletilla*.

## 2. Materials and Methods

### 2.1. Plant Materials

**Figure 1:**
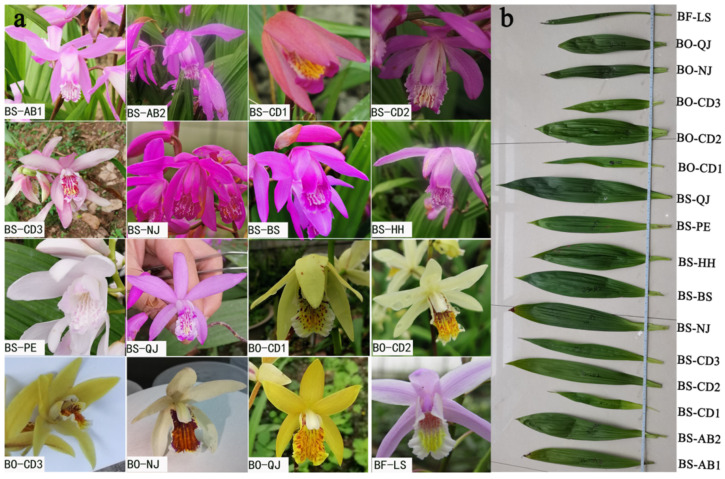
Morphology of sixteen samples in *Bletilla*. (**a**) Flower, (**b**) Leaf. BS-AB1: *B. striata* from Aba, Sichuan No 1; BS-AB2: *B. striata* from Aba, Sichuan No 2; BS-CD1: *B. striata* from Dujiangyan, Sichuan No 1; BS-CD2: *B. striata* from Pengzhou, Sichuan No 2; BS-CD3: *B. striata* from Chongzhou, Sichuan No 3; BS-NJ: *B. striata* from Neijiang, Sichuan; BS-BS: *B. striata* from Baoshan, Yunnan; BS-HH: *B. striata* from Honghe, Yunnan; BS-PE: *B. striata* from Pu’er, Yunnan; BS-QJ: *B. striata* from Qujing, Yunnan; BO-CD1: *B. ochracea* from Jingtang, Sichuan No 1; BO-CD2: *B. ochracea* from Dujiangyan, Sichuan No 2; BO-CD3: *B. ochracea* from Dujiangyan, Sichuan No 3; BO-NJ: *B. ochracea* from Neijiang, Sichuan; BO-QJ: *B. ochracea* from Qujing, Yunnan; BF-LS: *B. formosana* from Leshan, Sichuan.

Sixteen materials were collected from 2017 to 2018. Germplasm resources of *B. striata*, *B. formosana,* and *B. ochracea* were successively collected in Sichuan and Yunnan, China, as shown in Table 1. They were planted in the germplasm resources garden of the Chengdu Academy of Agriculture and Forestry Sciences. *B. formosana* had leaves that were thin and short, as well as small flowers. The leaves of *B. ochracea* were slender, and the flower type was medium. The flower type of *B. striata* was large, and the leaves were wide and thick. The phenological periods of the different varieties were obviously different. The germination time of *B. striata* was generally from February to March; for *B. formosana*, it was from February; and for *B. ochracea,* it was from March to April. In terms of the flowering period, *B. striata* blooms the earliest and begins to bloom successively around March, and has a shorter duration. *B. formosana* generally blooms in early May and has a longer duration. *B. ochracea* blooms the latest and has a longer flowering period, as shown in Figure 1a,b. 

### 2.2. Slaf Library Construction and Sequencing

DNA was extracted from the different samples using CTAB [43], and the diluted concentration samples were absorbed for nanodrop detection. As there was no information on the sequence of *Bletilla* that was released, the dual-digestion scheme was selected according to the genome size and GC content [24], followed by a dual-index sequencing joint reaction. The product was amplified with PCR and detected via electrophoresis and quantified via nanodrop. PCR-purified products were released from the library, and the obtained genetic data were identified, filtered, inspected, and evaluated using Nova-seq after sequencing to obtain the sequences of each *Bletilla* (reads) material for subsequent quality analysis [26]. In total, 16 read samples were identified from the original data using dual-index software, and the alleles were isolated. The quality of the read sequencing and data volumes were filtrated and analyzed. The enzyme digestion efficiency of the enzymes Rsa I and Hae III [44] was evaluated using control data to ensure the accuracy and validity of the data. The Rsa I and Hae III restriction endonucleases were used to cut enzyme fragments that were the same or similar in length from the 16 *Bletilla* samples. The reads were clustered according to similarity, and the reads that were clustered together were from the same SLAF label. Reference sequence development was based on genome-wide SNP markers that were of the sequence type, with the highest depth in each SLAF tag. Restriction endonuclease predictions, SLAF library construction, and library quality assessment were performed by Biomarker Technologies (Beijing, China).

### 2.3. FISH Hybridization

From 8:00 a.m. to 10:00 a.m. in the spring and autumn, when soil temperature was appropriate, 1~2 cm root tips were taken from the new growth meristematic zone of *Bletilla*. The root tips were cut and placed in a centrifugal tube, placed in 0.001 mol/L 8-hydroxyquinoline, and stored at 4 °C for 24 h, transferred into Carnoy for 4 °C for 24 h, and then had 70% alcohol added before being stored at −20 °C. The root tips were cleaned in ddH_2_O and then digested in cellulase and pectinase (4:2) enzymolysis solution, followed by water and anhydrous ethanol cleaning, and the addition of 20 μL glacial acetic acid drops on the slide. Then, the metaphase chromosomes were observed. The probes of the 5S rDNA sequences (41bp) [45] and (AG_3_T_3_)_3_ sequences (21 bp) [46] were synthesized by Sangon Biotech Co., Ltd. (Shanghai, China). The slides with a good splitting phase were hybridized with oligonucleotide sequences. After being cleaned and dried, DAPI was dropped and observed with an Olympus BX 63 (Olympus, Japan).

### 2.4. Data Analysis

Through bioinformatics analysis, SNP markers were developed using the sequence type with the highest depth in each SLAF tag as the reference sequence, and sequencing reads were compared with the reference genome using a Burrows–Wheeler Aligner (BWA) [47]. SNPs were developed using the Genome Analysis Toolkit (GATK) and SamTools [48], and the intersection of the SNP markers obtained with the two methods was used as the final reliable dataset of SNP markers. Genome-wide SNP markers were selected according to integrity > 0.5 and INF (integrity > 0.5) as the criteria for SNP site filtering and screening, and representative high-quality SNPs were screened for *Bletilla* and population heritability analysis. A phylogenetic tree was generated to describe the tree diagram of the phylogenetic relationship of the 16 materials; the distance matrix was constructed based on the genetic data, and the phylogenetic tree was based on the distance matrix. MEGA X [49] was used to construct the phylogenetic tree of Leucorrhea using a p-distance calculation model based on the neighbor-joining method. Bootstrap repeats were set 1000 times, revealing the taxonomic and evolutionary relationships among 16 *Bletilla* materials. The 16 samples could be divided into different subpopulations according to their gene frequencies, and there were different genetic distances among the different subpopulations. Based on the SNP location, admixture software [50] was used to analyze and study the population structure of the materials. At the same time, clustering was conducted according to the preset number of subgroups (K value), 1–10, and cross-validation clustering was carried out to determine the optimal number of clusters according to the minimum error rate. Analyzing the genetic structure of a population can intuitively quantify the possibility of population affinity clustering and ancestry origin. Principal component analysis (PCA) is a simplified statistical method for datasets. Through linear transformation, relevant variables are transformed into linearly unrelated variables, and the first large variance in data projection is made in the first coordinate system (PC1). The second variance is in the second coordinate system (PC2), etc. In this study, using the EIGENSOFT software [51], the genetic clustering of *Bletilla* populations was analyzed via principal component analysis. The FISH images were processed with Photoshop 2021 (Adobe Systems Incorporated, San Jose, CA, USA). They were named according to their length, statistical signal pattern, and karyotype data. (AG_3_T_3_)_3_ and 5S rDNA were used for labeling, wherein (AG_3_T_3_)_3_ was the red signal, and 5S rDNA was the green signal. The metaphase and prometaphase chromosomes of the three *Bletilla species* were analyzed with FISH. According to the relative length of the chromosomes, the ratio of ≥1.26 to the average length is L type; 1.25–1.01 is M2 type; 1.00–0.76 is M1 type; and ≤0.75 is S type.

## 3. Results

### 3.1. SLAF Tag Development

The total number of reads obtained from each sample was 2,034,833–7,385,716. The GC content was 38.93–41.41%, with an average of 39.98%, and the Q30 quality value of the sequenced bases was 90.38–94.86%, with an average value of 93.70%. The sequencing depth was 1,072,634–4,262,652, with an average value of 14.26X, indicating that the sequencing error rate was low and that the data obtained were reliable. A total of 484,764 SLAF tags were obtained from 16 *Bletilla* materials by means of sequence analysis, with an average sequencing depth of 14.26X. There were 283,972 polymorphic SLAF tags, with a total of 4,612,941 SNP markers. The SNP integrity was 5.87–14.82%, and the heterozygosity was 38.24–64.66%. Details are shown in Table 2.

### 3.2. Genetic Diversity Analysis

The SNP data based on mutation detection were filtered with the secondary allele frequency (MAF:0.05) and locus integrity (INT:0.8) to obtain high-conformance SNPs (661,139) for downstream analysis. The genetic relationships of 16 plant materials were evaluated using three methods.

**Figure 2:**
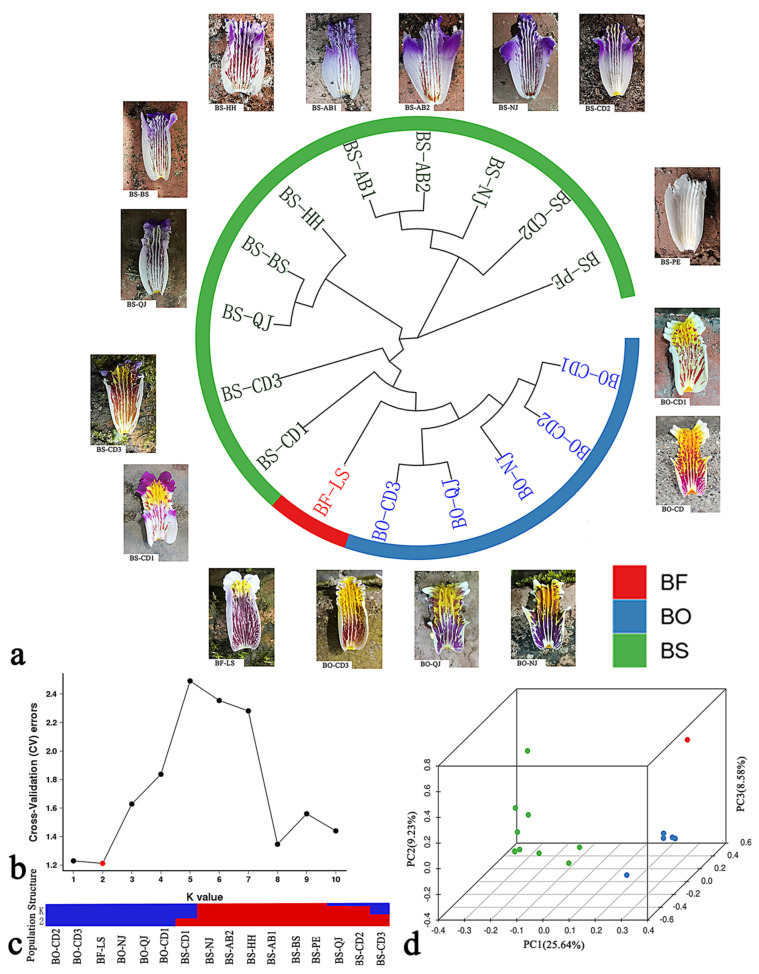
(**a**) Phylogenetic tree; (**b**) Cross-validation error rate of each k value of admixture; (**c**) Cross-validation error rate of each k = 2 value of admixture; (**d**) Three-dimensional cluster diagram (PC1, PC2, and PC3). BS-AB1: *B. striata* from Aba, Sichuan No 1; BS-AB2: *B. striata* from Aba, Sichuan No 2; BS-CD1: *B. striata* from Dujiangyan, Sichuan No 1; BS-CD2: *B. striata* from Pengzhou, Sichuan No 2; BS-CD3: *B. striata* from Chongzhou, Sichuan No 3; BS-NJ: *B. striata* from Neijiang, Sichuan; BS-BS: *B. striata* from Baoshan, Yunnan; BS-HH: *B. striata* from Honghe, Yunnan; BS-PE: *B. striata* from Pu’er, Yunnan; BS-QJ: *B. striata* from Qujing, Yunnan; BO-CD1: *B. ochracea* from Jingtang, Sichuan No 1; BO-CD2: *B. ochracea* from Dujiangyan, Sichuan No 2; BO-CD3: *B. ochracea* from Dujiangyan, Sichuan No 3; BO-NJ: *B. ochracea* from Neijiang, Sichuan; BO-QJ: *B. ochracea* from Qujing, Yunnan; BF-LS: *B. formosana* from Leshan, Sichuan.

MEGA X software was used to construct a phylogenetic evolutionary tree with a unique root node and a possible unique ancestor for 16 members of the genus *Bletilla*. The evolutionary tree was divided into four groups: The first group was the labellum color with the white-only phenotype; the second group included four copies of the materials from the Sichuan province varieties that were purple in color, flowered, and with a more prominent design and color; the third group included the varieties from Yunnan Province, with a larger flower type and a relatively large leaf size. All four groups included *B. formosana*, *B. ochracea,* and *B. striata* (safflower) or, more specifically, their common characteristic labellum with a yellow block. In terms of geographical location, the geographical distribution of *Bletilla* is correlated with the evolutionary tree to a certain extent. For example, the species of *Bletilla* growing in similar geographical locations are evolutionarily similar, but there is little correlation between *B. ochracea*, indicating that *B. ochracea* are highly mobile and are distributed and cross through Sichuan and Yunnan. As shown in Figure 2a, population structural analysis is a population cluster analysis method that is currently widely used. By analyzing the subgroups with different gene frequencies in the population, the relationship within the same subgroup is close, but the relationship between subgroups is distant. An admixture was used to analyze the population’s genetic results, and the error rate of the cross-validation clustering was the lowest when K = 2, as shown in Figure 2b,c. This may be related to the fact that only one case of *B. formosana* was collected in this experiment, and the samples were not representative. The 16 samples were divided into two subgroups. BO-CD2, BO-CD3, BF-LS, BO-NJ, BO-QJ, and BO-CD1 are probably derived from one ancestor, and 61.32% of BS-CD1 and 47.65% of BS-CD3 were derived from this ancestor, respectively. This branch was similar to the fourth group of phylogenetic trees, and the results could accurately distinguish the plants with middle yellow patches in the labellum. The first principal component (PC1), second principal component (PC2), and third principal component (PC3) explained 25.64%, 9.23%, and 5.58% of the genetic variation, respectively. The 16 materials could be divided into three nonoverlapping subgroups—namely, *B. formosana*, *B. ochracea,* and *B. striata*. The spatial distance reflected the genetic relationship. As shown in Figure 2d, which shows the PCA coordinates, PC1 represents the first principal component, etc. The coordinate space distance can be regarded as the judgment standard of kinship, as shown in Table 3.

### 3.3. Genetic Diversity Analysis of Bletilla Based on FISH

**Figure 3:**
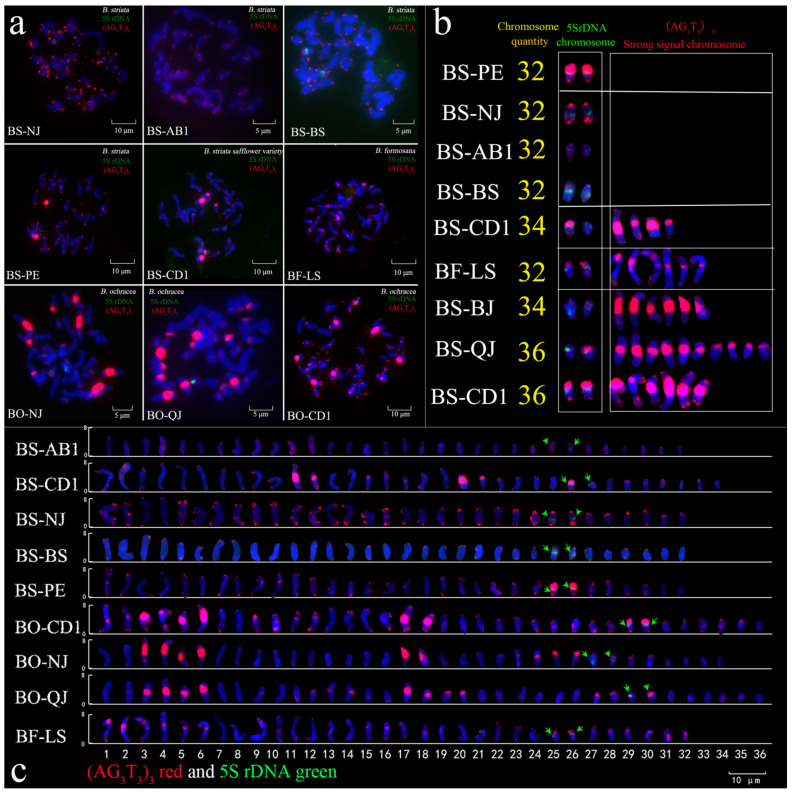
(**a**) Fluorescence in situ hybridization of *Bletilla*; (**b**) signal-mode difference diagram; (**c**) chromosomes vector graphs. Red represents 5S rDNA, while green represents (AG_3_T_3_)_3_. The blue chromosomes were counterstained with DAPI. Scale bar = 5 μm or 10 μm. BS-AB1: *B. striata* from Aba, Sichuan No 1; BS-CD1: *B. striata* from Dujiangyan, Sichuan No 1; BS-NJ: *B. striata* from Neijiang, Sichuan; BS-PE: *B. striata* from Pu’er, Yunnan; BS-BS: *B. striata* from Baoshan, Yunnan; BO-CD1: *B. ochracea* from Jingtang, Sichuan No 1; BO-QJ: *B. ochracea* from Qujing, Yunnan; BO-NJ: *B. ochracea* from Neijiang, Sichuan; BF-LS: *B. formosana* from Leshan, Sichuan.

Fluorescence in situ hybridization involved 9 out of the 16 materials and involved all the phenotypes and 3 varieties, as shown in Figure 3. Karyotype analysis is shown in Table 4. After repeated experiments using three root tips, the results were consistent. The number of chromosomes of *B. striata* and *B. formosana* was 2n = 32. The varieties of *B. striata* (safflower) had 34 chromosomes. The chromosome number of *B. ochracea* was 2n = 34~36. In terms of the number and width of chromosomes in different provenances of *Bletilla*, there were great differences in their length. For example, the total length difference between BS-NJ and BS-AB1 was 57.27 μm, and the difference between the length of *B. ochracea* was small. The staining length is related to the division stage and chromosome fixation method. Repeated experiments with three root tips yielded consistent results. The longest chromosome length was 8.54 μm, the shortest was 2.12 μm, the longest total length was 182.54 μm, and the shortest was 125.37 μm. When the chromosome length was larger, the difference between the *B. striata* chromosome length was smaller than that of *B. ochracea* and *B. formosana*, and the difference between the *B. formosana* chromosome length was the largest. This means that the degree of chromosome asymmetry was higher. The results indicate that the three species of *Bletilla* have higher degrees of asymmetry than other orchids, especially *B. ochracea* and *B. formosana*.

The combination patterns of the 5S rDNA signals and (AG_3_T_3_)_3_ signals were divided into five groups according to the signal pattern, as shown in Figure 2b. The first group was the one pair in which (AG_3_T_3_)_3_ had a strong signal location. Only the pure white labellum groups (BS-PE) were consistent with the first group of the SNP evolutionary tree. The second group, which had no (AG_3_T_3_)_3_ strong signal locus, represents BS-AB1, BS-BS, and BS-NJ, and was also consistent with the purple and yellow block group of the labellum group and groups 2 and 3 in the SNP evolutionary tree. Groups 3, 4, and 5 had strong (AG_3_T_3_)_3_ frequency zoning, which may be related to chromosome rearrangement and evolution. This region corresponds to the yellow labellum block group and group 4 in the SNP evolutionary tree. For example, in group 3, BS-CD1 had four non-5S rDNA signal chromosome-strong (AG_3_T_3_)_3_ signal loci; in group 4, it had relatively strong (AG_3_T_3_)_3_ signal loci; this group only had BS-LS; in group 5, it had multiple strong (AG_3_T_3_)_3_ signal loci, similar to BO-NJ, BO-CD1, BO-QJ, and another *B. ochracea*. The FISH karyotype analysis showed that there was abundant genetic diversity among nine materials from three species of *Bletilla*.

## 4. Discussion

### 4.1. Characteristics of the SLAF-seq Method

With the maturity and development of second-generation sequencing technology, determining the genome is simpler because of its easy operation, fast cycle, and low cost, and the reference base group does not need to be considered [52]. *Bletilla* has a complex genome, and until now, no whole-genome sequence information has been released for *Bletilla*. This article used SLAF dialogue-seq technology and a genome-wide SNP marker point to develop rich SNP marker loci. Many factors, such as climate, altitude, hydrology, and local human activities, can affect the genetic differentiation and population structure of *Bletilla*. Using admixture software dialogue and a natural population group structure, the analysis showed that the cross-minimum error rate was K = 2 based on *Bletilla*, and the classification pattern of the plant type could be divided into two classes. However, because only one sample was collected from *B. formosana*, we did not have a reference value for the classification, so the principal component analysis is consistent with the analysis structure for *B. striata* and *B. ochracea* classification [53]. There was also a certain correlation between the plants of different genera of *Bletilla* that were collected from similar areas, and the analysis results determining the population structure of the genera were more reliable [54]. The genetic relationships among individuals in different regions, flower colors, and genera were revealed, which provided a theoretical basis for the origin, differentiation, and genealogical geography of *Bletilla*. At the same time, it also provides a new approach and abundant molecular marker resources for molecular marker-assisted breeding, gene localization and selection breeding, variety identification, and protection. Subsequent studies can use the SNP loci information from this test to carry out further evolutionary analyses of the *Bletilla* population combined with epigenetic traits and the growth cycle to localize excellent genes to aid in gene-editing-oriented breeding [55]. The results of the phylogenetic tree and principal component analysis were consistent, and compared with the signal pattern grouping of the fluorescence in situ hybridization and the morphology grouping of the apparent labellum, the explanation and the results of the genus population structural analysis were more reliable.

### 4.2. Differences in Karyotype Structure and FISH Signal Pattern

Karyotype analysis was used to reveal the genome of the chromosome-level information. Karyotypes provide the cytological features [56] to determine the origin of species, develop information systems, genetic breeding, and variety improvements [57], and provide the basis for the naming of plants. The karyotypes of *Bletilla* have been less studied. At present, only Wang [58] has carried out karyotype analysis on *B. striata* and has obtained the karyotype formula. The karyotype of Orchidaceae is relatively complex [59]. The number of other orchids is more complex and is 2n = 42 in *Crepidium* Blume, 2n = 26 in *Paphiopedilum* Pfitzer, and 2n = 38–44 in *Pleione* D. Don [60,61]. In the karyotype structure, the dyeing length is divided by the number of chromosomes, and the chromosome method is fixed. The differences in the chromosome length of *B. striata* are less than those in *B. formosana* and *B. ochracea,* and the greater the difference between the *B. formosana* chromosomes, the higher the degree of asymmetry in the plant chromosome, reflecting that its evolution or specialized degree is higher compared, with the material and the original [62]. The degree of asymmetry in the three species of *Bletilla* is higher, especially in *B. formosana* and *B. ochracea*. In addition, the number of *B. striata* (safflower) is between that of *B. striata* and *B. ochracea*, and the labellum pattern is more similar to *B. ochracea* and may be formed by natural hybridization between the two species. In terms of regional distribution, multiple locations were considered in Sichuan and Yunnan provinces. In addition to the one sample of *B. formosana* that was collected, three samples were collected for both *B. striata* and *B. ochracea*. In all the groups, there are differences between habitats, and distance also causes differentiation between groups. The greater the ecological environment differences are, the more unique traits, such as color, leaf type, disease and insect resistance, and the content of tuber polysaccharides determined by natural selection formed in the long term. The direct reflection of these traits on the chromosome may be missing. Translocation and inversion structure variation, such as major rearrangements, and divergence in the chromosome structure may make adaptation more obvious [61] because the centromere is not obvious, and the karyotype and cell type have been curbed. This test is not suitable for further karyotype analysis [45]. The differences in the karyotype analysis results reflect the high diversity of genetic resources in *B. striata*, *B. formosana,* and *B. ochracea*.

(AG_3_T_3_)_3_ signals are usually detected at terminal positions, with occasional signals detected around and inside the centromeres, and have been verified in plants belonging to the genera *Hydrocerus*, *Syringa*, and *Ligustrum* [63]. (AG_3_T_3_)_3_ probe signals can be found in all chromosomes and show that the chromosome shape is complete. In *Bletilla,* the size of the (AG_3_T_3_)_3_ signal in the genus is different. In some samples, the end signal of (AG_3_T_3_)_3_, may be related to the chromosome rearrangement structure. *B. ochracea* has a high degree of chromosome rearrangement and a stronger signal site. The 5S rDNA signal binds around the ribosome and is conducive to chromosome differentiation [64]. This is determined according to the signal site and strength of the phase, such as in *Piptanthus concolor* Harrow ex Craib [45]. In addition, the number of signal loci in some species can reflect the chromosome ploidy [65], which is conducive to reproductive separation and speciation [66] and chromosome characterization [63]. All the varieties of *Bletilla* had two rDNA signals, consistent with previous results for other *Bletilla* species, indicating that *Bletilla* are diploids and that different species have different signal intensities. The combination of (AG_3_T_3_)_3_ and 5S rDNA has been verified in plant species such as *C.campanulatus* [39] and *H. rhamnoides* [38]. However, compared with other plants, the (AG_3_T_3_)_3_ signals of *B. formosana* and *B. ochracea* were unstable, which may be related to the degree of evolution. In addition, the non-terminal signal of (AG_3_T_3_)_3_ with the labellum with a characteristic yellow block was strong, similar to the differentiation of the SNP evolutionary tree, indicating that this type of material has the same ancestor and evolutionary trend and can be divided into separate groups. SNP, oligo-FISH signal pattern, and the phenotypic labellum can verify each other. Therefore, the experiment has certain accuracy.

### 4.3. SNP, FISH, and Parental Breeding

FISH data reflect the ploidy and karyotype relationship of *Bletilla*, providing evidence for ploidy breeding and variety identification [67]. The molecular markers of SNPs are polymorphisms caused by deletion, insertion, conversion, and other mutations of a single nucleotide, and there are a large number of SNP polymorphic sites in the genome [68]. SNP genetic analysis can effectively judge how closely related different *Bletilla* varieties are. SNP sequencing cannot distinguish between *B. striata* and *B. formosan* and is able to distinguish them at the chromosome level. Chromosome data can be used to study species evolution, classification, and breeding [69]. It is scientific, possible, and accurate to classify *B. striata* and *B. formosan* at the chromosome level. The lack of differentiation at the molecular level may be related to the evolution of *B. formosan*, which is reflected in the labellum valve phenotype. At the same time, due to the great depth of sequencing, it is difficult to cluster the results. Of course, these results may also be related to sampling errors and their test data. Combined with population genetic analysis to determine the SNP molecular markers, the fluorescence in situ hybridization results for *Bletilla* have a certain degree of correlation with SNP population genetic analysis, showing the polymorphism differences on the basis of cytogenetics, which also provide a basis for the differentiation of *Bletilla.* Improved varieties are often selected for higher phenotypic variation [70] or because they have a more distant genetic relationship [71] to improve the breeding efficiency of special varieties. Both FISH and SNP can be used to analyze the genetic diversity of *Bletilla* varieties and to understand the genetic backgrounds of the bred varieties, laying a foundation for parental selection in future *Bletilla* breeding projects [72].

## 5. Conclusions

Through population genetic analysis, it was found that *Bletilla* shows rich polymorphism in different populations and that (AG_3_T_3_)_3_ and 5S rDNA can effectively distinguish three species of *Bletilla* via their signals.

## Figures and Tables

**Table 1:** Collection of germplasm resources.

Sample	Variety	Location	Geographic Coordinates	Geographic Coordinates	Leaf Spreading	Initial Flowering	Green Period	Florescence
BS-AB1	*B. striata*	Aba, Sichuan	103°37′30″ E, 31°28′05″ N	1529 m	04, Dec.	15, Mar.	316 d	41 d
BS-AB2	*B. striata*	Aba, Sichuan	103°23′59″ E, 30°55′55″ N	1348 m	21, Feb.	11, Mar.	229 d	47 d
BS-CD1	*B. striata*	Dujiangyan, Sichuan	103°40′19″ E, 31°06′36″ N	1389 m	21, Feb.	01, Apr.	235 d	39 d
BS-CD2	*B. striata*	Pengzhou, Sichuan	103°55′51″ E, 31°08′51″ N	690 m	11, Feb.	25, Mar.	238 d	28 d
BS-CD3	*B. striata*	Chongzhou, Sichuan	103°43′48″ E, 30°40′46″ N	543 m	21, Feb.	01, Apr.	235 d	39 d
BS-NJ	*B. striata*	Neijiang, Sichuan	104°53′54″ E, 29°35′11″ N	365 m	09, Feb.	15, Mar.	247 d	48 d
BS-BS	*B. striata*	Baoshan, Yunnan	99°16′28″ E, 25°16′54″ N	2320 m	11, Mar.	15, Apr.	211 d	44 d
BS-HH	*B. striata*	Honghe, Yunnan	102°17′25″ E, 23°16′29″ N	2014 m	14, Feb.	15, Mar.	236 d	55 d
BS-PE	*B. striata*	Pu’er, Yunnan	101°05′22″ E, 23°00′18″ N	1406 m	14, Feb.	01, Apr.	229 d	55 d
BS-QJ	*B. striata*	Qujing, Yunnan	103°48′24″ E, 25°40′35″ N	1996 m	05, Mar.	19, Apr.	217 d	27 d
BO-CD1	*B. ochracea*	Jintang, Sichuan	104°29′22″ E, 30°53′59″ N	628 m	09, Mar.	25, Apr.	206 d	69 d
BO-CD2	*B. ochracea*	Dujiangyan, Sichuan	103°40′37″ E, 31°09′20″ N	1119 m	01, Apr.	25, Apr.	190 d	73 d
BO-CD3	*B. ochracea*	Dujiangyan, Sichuan	103°39′41″ E, 31°06′28″ N	1145 m	06, Mar.	28, Apr.	209 d	61 d
BO-NJ	*B. ochracea*	Neijiang, Sichuan	104°51′51″ E, 29°35′46″ N	405 m	25, Feb.	23, Apr.	225 d	132 d
BO-QJ	*B. ochracea*	Qujing, Yunnan	103°25′25″ E, 26°27′17″ N	2067 m	01, Apr.	09, May	183 d	54 d
BF-LS	*B. formosana*	Leshan, Sichuan	103°54′38″ E, 28°56′42″ N	468 m	09, Feb.	09, May	249 d	135 d

Note: Leaf spreading and initial flowering show the DATE.

**Table 2:** SLAF tag statistics for the samples and SNP information statistics.

Sample	Total Reads	SLAF Number	Total Depth	SNP Number	Hetloci Ratio (%)	Integrity Ratio (%)	GC (%)	Q30 (%)
BS-AB1	3,821,883	170,860	2,473,500	2,276,348	6.61	49.34	39.38	93.80
BS-AB2	4,812,117	179,144	3,064,920	2,455,325	7.35	53.22	38.94	93.99
BS-CD1	5,141,345	193,721	2,434,635	2,754,599	12.84	59.71	41.28	94.08
BS-CD2	5,981,956	206,013	2,965,498	2,965,366	11.13	64.28	40.44	92.85
BS-CD3	5,784,414	223,459	3,307,779	2,982,996	14.82	64.66	41.22	94.58
BS-NJ	2,949,503	158,898	1,783,602	2,136,444	5.87	46.31	39.15	93.40
BS-BS	3,997,451	176,902	2,613,096	2,283,183	9.87	49.49	39.35	93.89
BS-HH	2,034,833	145,657	1,072,634	1,764,431	6.56	38.24	38.74	94.06
BS-PE	3,061,044	158,054	1,839,338	2,095,856	4.35	45.43	39.74	93.82
BS-QJ	7,385,716	218,414	4,061,827	2,889,780	12.62	62.64	40.63	93.71
BO-CD1	2,573,939	140,044	1,747,803	1,670,758	7.77	36.21	38.93	93.59
BO-CD2	5,337,341	185,699	2,831,409	2,399,920	12.09	52.02	39.15	90.38
BO-CD3	4,403,717	173,407	2,934,318	2,099,281	8.92	45.50	41.41	94.00
BO-NJ	7,037,001	216,078	4,262,652	2,892,493	11.51	62.70	40.89	94.58
BO-QJ	2,932,721	149,933	1,776,647	1,891,899	7.75	41.01	39.02	93.65
BF-LS	4,799,485	173,473	2,605,232	2,468,689	10.40	53.51	41.39	94.86

Note: BS-AB1: *B. striata* from Aba, Sichuan No 1; BS-AB2: *B. striata* from Aba, Sichuan No 2; BS-CD1: *B. striata* from Dujiangyan, Sichuan No 1; BS-CD2: *B. striata* from Pengzhou, Sichuan No 2; BS-CD3: *B. striata* from Chongzhou, Sichuan No 3; BS-NJ: *B. striata* from Neijiang, Sichuan; BS-BS: *B. striata* from Baoshan, Yunnan; BS-HH: *B. striata* from Honghe, Yunnan; BS-PE: *B. striata* from Pu’er, Yunnan; BS-QJ: *B. striata* from Qujing, Yunnan; BO-CD1: *B. ochracea* from Jingtang, Sichuan No 1; BO-CD2: *B. ochracea* from Dujiangyan, Sichuan No 2; BO-CD3: *B. ochracea* from Dujiangyan, Sichuan No 3; BO-NJ: *B. ochracea* from Neijiang, Sichuan; BO-QJ: *B. ochracea* from Qujing, Yunnan; BF-LS: *B. formosana* from Leshan, Sichuan.

**Table 3:** Cluster correspondence of PCA.

Variety	BS- NJ	BS- AB1	BS- BS	BS- PE	BS- CD1	BF- LS	BO- NJ	BO- QJ	BO- CD1	BS- AB2	BS- QJ	BS- CD2	BS- CD3	BO- CD2	BS- HH	BO- CD3
PC1	−0.2613	−0.2832	−0.2481	−0.2597	0.0845	0.2604	0.3081	0.3191	0.273	−0.2832	−0.1605	−0.1813	0.0031	0.3032	−0.1995	0.3252
PC2	−0.1949	−0.2126	0.1878	−0.0381	0.0558	0.4205	−0.1298	−0.0525	−0.1376	−0.2185	0.7152	−0.2128	−0.1489	−0.0407	0.1325	−0.1254
PC3	0.0893	0.1009	−0.0284	0.0486	−0.3773	0.5683	0.1491	−0.5937	0.1518	0.1037	−0.1932	0.0665	−0.2157	0.0329	−0.0248	0.122

Note:BS-AB1: *B. striata* from Aba, Sichuan No 1; BS-AB2: *B. striata* from Aba, Sichuan No 2; BS-CD1: *B. striata* from Dujiangyan, Sichuan No 1; BS-CD2: *B. striata* from Pengzhou, Sichuan No 2; BS-CD3: *B. striata* from Chongzhou, Sichuan No 3; BS-NJ: *B. striata* from Neijiang, Sichuan; BS-BS: *B. striata* from Baoshan, Yunnan; BS-HH: *B. striata* from Honghe, Yunnan; BS-PE: *B. striata* from Pu’er, Yunnan; BS-QJ: *B. striata* from Qujing, Yunnan; BO-CD1: *B. ochracea* from Jingtang, Sichuan No 1; BO-CD2: *B. ochracea* from Dujiangyan, Sichuan No 2; BO-CD3: *B. ochracea* from Dujiangyan, Sichuan No 3; BO-NJ: *B. ochracea* from Neijiang, Sichuan; BO-QJ: *B. ochracea* from Qujing, Yunnan; BF-LS: *B. formosana* from Leshan, Sichuan.

**Table 4:** Karyotype analysis of sixteen samples in *Bletilla*.

Samples	Species	Total Length	Karyotype Formula	Longest Chromosome	Shortest Chromosome
BS-NJ	*B. striata*	174.85 μm	2n = 32 = 6L + 9M2 + 8M1 + 9S	8.28 μm	3.26 μm
BS-AB1	*B. striata*	117.53 μm	2n = 32 = 8L + 7M2 + 12M1 + 5S	5.54 μm	2.12 μm
BS-BS	*B. striata*	147.06 μm	2n = 32 = 5L + 10M2 + 11M1 + 6S	7.10 μm	2.49 μm
BS-PE	*B. striata*	160.06 μm	2n = 32 = 7L + 7M2 + 9M1 + 9S	8.10 μm	2.24 μm
BS-CD1	*B. striata*	166.84 μm	2n = 34 = 10L + 5M2 + 7M1 + 12S	7.55 μm	2.24 μm
BF-LS	*B. formosana*	179.26 μm	2n = 32 = 10L + 3M2 + 10M1 + 9S	8.54 μm	2.85 μm
BO-NJ	*B. ochracea*	151.97 μm	2n = 34 = 7L + 10M2 + 9M1 + 8S	7.85 μm	2.86 μm
BO-QJ	*B. ochracea*	147.31 μm	2n = 36 = 11L + 2M2 + 15M1 + 8S	6.61 μm	2.46 μm
BO-CD1	*B. ochracea*	182.54 μm	2n = 36 = 9L + 6M2 + 11M1 + 10S	8.69 μm	2.75 μm

Note: BS-AB1: *B. striata* from Aba, Sichuan No 1; BS-CD1: *B. striata* from Dujiangyan, Sichuan No 1; BS-NJ: *B. striata* from Neijiang, Sichuan; BS-PE: *B. striata* from Pu’er, Yunnan; BS-BS: *B. striata* from Baoshan, Yunnan; BO-CD1: *B. ochracea* from Jingtang, Sichuan No 1; BO-QJ: *B. ochracea* from Qujing, Yunnan; BO-NJ: *B. ochracea* from Neijiang, Sichuan; BF-LS: *B. formosana* from Leshan, Sichuan.

## Data Availability

All data and materials are included in the form of graphs in this article.
